# Protocol for a randomised controlled trial for Reducing Arthritis Fatigue by clinical Teams (RAFT) using cognitive–behavioural approaches

**DOI:** 10.1136/bmjopen-2015-009061

**Published:** 2015-08-06

**Authors:** S Hewlett, N Ambler, C Almeida, P S Blair, E Choy, E Dures, A Hammond, W Hollingworth, J Kirwan, Z Plummer, C Rooke, J Thorn, K Tomkinson, J Pollock

**Affiliations:** 1 Department of Nursing and Midwifery, University of the West of England Bristol, Bristol, UK; 2 Pain Management Centre, Southmead Hospital, Bristol, UK; 3 School of Social and Community Medicine, University of Bristol, Bristol, UK; 4 Section of Rheumatology, Institute of Infection and Immunity, Cardiff University, Cardiff, UK; 5 Centre for Health Sciences Research, School of Health Sciences, University of Salford, Salford, UK; 6 Academic Rheumatology, School of Clinical Sciences, University of Bristol, Bristol, UK; 7 Patient research partner, Academic Rheumatology, Bristol Royal Infirmary, Bristol, UK; 8 Department of Health and Social Sciences, University of the West of England Bristol, Bristol, UK

**Keywords:** RHEUMATOLOGY

## Abstract

**Introduction:**

Rheumatoid arthritis (RA) fatigue is distressing, leading to unmanageable physical and cognitive exhaustion impacting on health, leisure and work. Group cognitive–behavioural (CB) therapy delivered by a clinical psychologist demonstrated large improvements in fatigue impact. However, few rheumatology teams include a clinical psychologist, therefore, this study aims to examine whether conventional rheumatology teams can reproduce similar results, potentially widening intervention availability.

**Methods and analysis:**

This is a multicentre, randomised, controlled trial of a group CB intervention for RA fatigue self-management, delivered by local rheumatology clinical teams. 7 centres will each recruit 4 consecutive cohorts of 10–16 patients with RA (fatigue severity ≥6/10). After consenting, patients will have baseline assessments, then usual care (fatigue self-management booklet, discussed for 5–6 min), then be randomised into control (no action) or intervention arms. The intervention, Reducing Arthritis Fatigue by clinical Teams (RAFT) will be cofacilitated by two local rheumatology clinicians (eg, nurse/occupational therapist), who will have had brief training in CB approaches, a RAFT manual and materials, and delivered an observed practice course. Groups of 5–8 patients will attend 6×2 h sessions (weeks 1–6) and a 1 hr consolidation session (week 14) addressing different self-management topics and behaviours. The primary outcome is fatigue impact (26 weeks); secondary outcomes are fatigue severity, coping and multidimensional impact, quality of life, clinical and mood status (to week 104). Statistical and health economic analyses will follow a predetermined plan to establish whether the intervention is clinically and cost-effective. Effects of teaching CB skills to clinicians will be evaluated qualitatively.

**Ethics and dissemination:**

Approval was given by an NHS Research Ethics Committee, and participants will provide written informed consent. The copyrighted RAFT package will be freely available. Findings will be submitted to the National Institute for Health and Care Excellence, Clinical Commissioning Groups and all UK rheumatology departments.

**Trial registration number:**

ISRCTN: 52709998; Protocol v3 09.02.2015.

**Strengths and limitations of this study**

- A pragmatic study testing a cognitive-behavioural intervention that could be delivered by the usual care team.
- A seven-centre trial targeting a broad population, thus, results likely to be widely generalisable.
- Patient impact is evaluated in a holistic fashion (eg, including valued life activities) alongside health economic evaluation.
- This pragmatic trial includes usual care throughout; any medication changes have the potential to alter fatigue and, therefore, the trial results (if changes differ between arms).

## Introduction

Rheumatoid arthritis (RA) is a systemic inflammatory condition causing synovitis in multiple joints, pain, joint destruction and disability.^1^ Life-long treatment is by secondary care rheumatology teams, using medication to control inflammation and multidisciplinary interventions to reduce symptoms and maximise self-management.^2^ ^3^ RA affects approximately 500 000 people in the UK,^4^ and fatigue is present on most days for most people, with >70% reporting levels similar to Chronic Fatigue Syndrome.^5^ ^6^ RA fatigue is overwhelming physical exhaustion that impacts on social and work activities, and has cognitive and emotional elements: poor concentration, frustration and tearfulness.^7^ ^8^ People with RA identify fatigue as the main reason for work loss, which affects >60% of working patients, with production loss estimated at >£650 million.^9–12^ Fatigue predicts and reduces quality of life, is as hard to cope with as pain; patients rate fatigue as a top priority,^13–16^ and international consensus states it must be evaluated in all RA clinical trials.^17^

Patients consider this unmanageable symptom as ignored by clinicians,^7^ and systematic review shows biological agents for RA inflammation have only a small effect on fatigue.^18^ RA fatigue is associated with inflammation, pain, disability, sleep, depression and beliefs,^19–21^ implying complex, multicausal pathways comprising differing combinations of variables.^22^ ^23^ This highlights the critical need for self-management interventions. Systematic review of randomised controlled trials (RCTs) of non-pharmacological interventions demonstrates moderate but significant effects of exercise, and of cognitive–behavioural therapy (CBT) on RA fatigue.^24^

CBT helps patients understand the thoughts and feelings that drive behaviours, and uses cognitive restructuring to help them make behaviour changes.^25^ For example, a belief that tasks must be done perfectly, and feeling guilty if they are not, may drive episodes of excessive activity, leading to episodes of enforced rest (‘boom and bust’). In CBT, key self-management skills of problem-solving and goal-setting can be enhanced by sharing the learning process in groups with other patients as role models (social cognition theory; SCT) to increase self-efficacy, or confidence that you can do something.^26^ Systematic reviews conclude that rheumatology self-management courses using CBT and/or SCT are more effective than information alone.^27^ ^28^ Three RCTs using group education, individual CBT or group CBT reported reduction in RA fatigue.^29–31^ However, fatigue was never the primary aim, entry criteria did not include fatigue and was selective (early disease, low disability, high distress). As RA fatigue is commonplace, self-management interventions should target the broad RA population. Therefore, in 2007–2009, an RCT conducted by this group using broad RA inclusion criteria and fatigue ≥6/10, compared a group CBT fatigue self-management programme with information alone.^32^ Group CBT improved fatigue impact (effect size 0.77), severity and coping, disability, depression, sleep, helplessness and self-efficacy. The intervention was co-facilitated by a clinical psychologist and pain management Occupational Therapist (OT), and in the nested qualitative study, patients spontaneously raised CBT elements as key to its success.^33^ However, few rheumatology units have clinical psychologists, thus, the programme cannot currently be routinely delivered in clinical practise. The same issue arises for multiple sclerosis and other long-term conditions (LTC) where CBT reduces fatigue.^34^ Thus, for LTC services, if the usual clinical teams could deliver an effective cognitive–behavioural (CB) intervention for fatigue, it could be embedded in usual care, delivered by clinicians who routinely support patients in self-management, and understand how fatigue interacts with disease fluctuations, pain and disability. Self-management of LTCs and improving access to psychological therapies are key government targets;^35–37^ while RA-specific guidelines by National Institute for Health and Care Excellence (NICE)^38^ and other professional bodies^2^ ^3^ recommend support for self-management, fatigue, and use of CB therapies. A search of RCT registration databases found no trials of CB interventions by the usual clinical team for RA fatigue.

The primary aim of this RCT is to assess whether there is a clinically important difference in the impact of fatigue between a group CB self-management course for RA fatigue delivered by two members of the usual clinical rheumatology team in addition to usual care; compared to usual care alone (information booklet). The secondary aims are to: (1) compare differences between groups for fatigue severity, fatigue coping, mood, sleep, helplessness, pain, disability, valued activities, quality of life, work, health service use, acceptability and cost-effectiveness for the National Health Service (NHS), patients and society; (2) evaluate and control for potential demographical, psychological and clinical predictors of fatigue change; (3) evaluate persistence of effect over 2 years and (4) explore whether clinical teams trained in CB approaches perceive any positive or negative outcomes, particularly on their wider clinical practise. Clinical rheumatology teams will be trained to deliver Reducing Arthritis Fatigue by clinical Teams (RAFT) using CB approaches, which is a manualised version of the original CBT intervention.^32^

## Methods

### Study design

To test generalisability for wide delivery across the NHS, this RCT involves seven rheumatology units encompassing large/small, academic/non-academic departments in city/rural areas. Blinding of patients and clinicians is not possible because of the need to engage patients in making cognitive and behavioural changes. However, outcomes are obtained using validated patient self-report measures, and analysis will be performed blind to treatment allocation.

### Sample selection and recruitment procedures

Inclusion criteria are patients aged ≥18 years with a confirmed diagnosis of RA^39^ and fatigue severity ≥6/10 on a numerical rating scale (NRS),^40^ which they consider persistent or recurrent, thereby targeting a broad RA population. Exclusion criteria are insufficient English language to participate in group discussions, lacking capacity for informed consent or recent changes to major RA medication (16 weeks) or glucocorticoids (6 weeks).

A research nurse in each centre will aim to approach all patients with RA attending for outpatient appointments, and may send mailshots using departmental databases. Interested patients will complete a screening fatigue NRS, and other eligibility criteria will be checked. If a patient is interested but not currently eligible, the nurse will rescreen them after 3–4 months. The anonymised age, gender and fatigue NRS of those who decline will be recorded.

The nurse will discuss the patient information sheet with eligible patients and inform them of the dates for the next CB course (highlighting that they may/may not be randomised to receive it). Over a period of 2 years, each centre will recruit four consecutive cohorts of 10–16 patients. Closure of recruitment to a cohort will be agreed with RAFT's central study team. The research nurse will invite patients to attend for written consent, baseline assessments and ‘usual care’. The visits will take place over a 2-week period, and when all are completed, that cohort will be randomised. Building each cohort will take several months; therefore, to maintain engagement, the nurse will call waiting patients monthly to update them.

### Randomisation and allocation concealment

The research nurse will give each patient the next unique study identifier (ID) immediately after written consent. As patients are offered a choice of baseline visit dates/times, it is unlikely that ID allocation can be influenced. Whenever a centre has completed the baseline visits for a cohort of 10–16 participants, the central RAFT Trial Manager will email the ID list to Bristol Randomised Trials Collaboration (BRTC) who will manage the computer-generated randomisation using an access database. Randomisation will be stratified by centre and within each centre, by cohort (cohorts 1–4). Allocation will be 1:1, but in the event of an odd number, the CB arm will receive the additional patient. Upon receiving the arm allocations from Bristol, the research nurse will inform the consented participants by telephone, following guidelines to ensure a neutral conversation (ie, no implied potential benefit from a CB allocation), reminding participants they have already received usual care at baseline.

Patients randomised to the CB course but who are unable to attend the next course dates, will be offered a subsequent course, with a new baseline evaluation. Any changes to eligibility between screening and baseline (eg, fatigue less severe, medication change) will be noted for subgroup analysis, but the patient will proceed to randomisation. This reflects the pragmatic nature of delivering interventions to a population with recurrent but fluctuating fatigue.

### Sample size

The baseline-adjusted effect size (0.77) for fatigue impact found in the original trial of CBT delivered by a clinical psychologist was 1.95 units (SD=2.7, 0–10 visual analogue scale).^32^ The current RCT of CB approaches delivered by rheumatology teams is powered to be able to demonstrate an average effect size across all participating centres of 75% of this (1.46 units, effect size 0.54)—clinically important, as it would reduce the number of patients falling below the fatigue criterion for trial entry by one-third. There are no published data on a minimal clinically important difference (MCID) for RA fatigue impact, but MCID for RA fatigue severity is 0.82–1.12 (0–10 scale).^41^ At a power of 90% and two-sided significance of 0.05, this requires 73 patients/arm. As we are interested in the average effect across all centres, we expect no loss of power as a result of randomising patients by site.^42^ ^43^

There are two potential sources of clustering in the intervention arm: CB group effects and centre/tutor effects. In the original trial,^32^ the intraclass correlation coefficient (ICC) for the CBT group using the primary outcome was an estimated <0.00 001. No data exist for centre/tutor effects of CBT on RA fatigue, so we are taking an ICC value of 0.01 for groups clustered within centres.^44^ The resulting design effects increase the required sample size to 75/arm.

In the original RCT^32^ most attrition occurred after patients had completed their intervention, that is, loss to questionnaire completion. To minimise this, the primary outcome (fatigue impact, NRS) will be collected by telephone by the central RAFT team, missing data returns will be followed up, and patients who wish to withdraw will be asked if they would provide just their fatigue impact NRS. We anticipate obtaining 80% of primary outcome data (26 weeks). Longer term attrition is unknown, therefore, we have assumed 50% and a planned capacity to recruit up to 150 patients/arm, to maintain sufficient power at 2 years. A pragmatic approach to recruitment is needed to take account of natural variations in group size. Therefore, a target of seven centres, each running four CB courses with an average of six participants/group (n=168 in CB arm) provides contingency for some smaller groups.

### Intervention, tutor training and supervision

*Intervention*: RAFT development followed the MRC framework for complex interventions.^45^ RAFT is delivered to groups of 5–8 patients with RA, in 6×2 h sessions (weeks 1–6) and a 1 h consolidation session (week 14). Sessions are cofacilitated by two members of the rheumatology team (eg, nurse, OT) who have been trained in RAFT (tutors).

RAFT content encapsulates the complex conceptual framework of RA fatigue,^22^ in which thoughts, feelings and behaviours interact with disease processes and consequences (eg, inflammation, disability), and personal context (values, circumstances) to exacerbate or perpetuate fatigue. The first hour of each session is a whole-group discussion facilitated by the tutors. After a 15 min break, the tutors divide the group into two, allowing each patient to discuss their goals within a smaller group. Topics build on each other week by week (table 1). Homework, activity diaries and goal setting are classic CB approaches.^25^

**Table 1:** RAFT course design

Week	First hour	Supporting materials*	Second hour
1	Course purpose and expectations Ground rules: Commitment, confidentiality, homework Validating fatigue: Share and discuss fatigue experiences (difference from flare) Self-management strategies, struggles and difficulty of changing habits	H: Arthritis Research UK booklets H: Setting our course (groups’ ideas)	Energy management Boom and bust behaviour Rewards/pitfalls of this Prioritise, pace, plan, choicesH: Achieving balance H: Activity cycling T: Activity/rest diaries
2	What are your priorities for change, to ↑QoL? What are your drainers and energisers?	T: Wheel of life (priority areas)	Goal setting (two groups) Short-term/long-term goals Use peer group for ideas
3	Self-sabotage on the course Sleep and rest Hours needed? Quality vs quantity Sleep hygiene strategies	H: Best ways of self-sabotage H: Getting a better night's sleep T: Sleep diary (if needed)	Goal-setting review Successes/barriers New goals
4	Stress and relaxation Personal stressors, bodily reactions Relaxation rationale and techniques	H: Effects of stress H: Relaxation practice guide T: Relaxation CD	Goal-setting review Successes/barriers New goals
5	Assertiveness and communication Passive, manipulative, assertive? Other people's reactions to these? Communicating your needs	M: Cartoon examples H: Saying ‘No’	Goal-setting review Successes/barriers New goals
6	Review self-help tools What have you learnt? Review each topic Dealing with setbacks—what could you do? Negative self-talk, automatic thoughts, rumination	M: Fatigue pit: Falling in/digging out H: The pit; Coping with setbacks	Goal-setting review Successes/barriers New goals
14	Review last 8 weeks; skills; dealing with setbacks; new goals	M: Islands: Was on Desert island (passive) looking at the Mainland (100% healthy, unrealistic). Now on Adaptive Coping Island (realistic)	

*H, handouts; M, metaphor; RAFT, Reducing Arthritis Fatigue by clinical Teams; T, tools.

Week 1 starts with discussions that validate fatigue experiences and the struggle to self-manage. After coffee, tutors draw ideas from patients on why they persist in boom and bust behaviours (eg, the rewards of getting things done), and the discussion is steered towards helping patients generate positive strategies for energy management (eg, prioritising, pacing). This is built on by the patients’ homework, which is to self-monitor their activity, rest and fatigue patterns by colouring in a daily activity/rest chart every hour for the week. In week 2, the discussion explores personal priorities, and moves into goal setting. Here, the small groups review each person's daily activity chart to understand patterns of behaviour and their consequences (fatigue), and each patient is encouraged to set personal short and long-term goals, using their activity/rest chart to help them identify potential behavioural changes (typically reducing boom and bust behaviours). These charts are the focus of the small group discussions throughout RAFT, and as they are completed each week they should show an improving balance of rest/activity. Review of the latest activity charts is related to sleep and rest (week 3), which link to relaxation and stress (week 4), and difficulty communicating their needs (week 5). The skills introduced in weeks 1–5 are reviewed in week 6, and tutors draw from patients how to deal with setbacks (metaphor of being in a pit). The seventh session (week 14, after patients have been trying new behaviours for 8 weeks) reflects on self-management progress (metaphor of leaving a desert island), consolidates the skills learnt, reflects on how those worked in the real world, and helps patients set future goals.

In the case of unexpected tutor absence (eg, illness), the remaining tutor will either deliver the session alone, delay by 1 week, or ask another clinical team member to attend as a supporter. This clinician will not deliver course content, and the group will not split for goal setting. If there is long-term tutor absence, a new tutor will be trained. If a patient is unable to attend a session, they will be invited to come early the next week for tutors to explain key content that was missed.

*Tutor training*: The RAFT manual contains detailed instructions for each of the seven sessions, including the key points to be drawn from patients, sample conversations, suggested timings, and all required materials (handouts, metaphors, goal-setting records, activity charts) with a clear indication of when and how they are to be used. The seven pairs of tutors will be trained together in Bristol over four consecutive days by the psychologist (Ambler) and OT (Knops) who delivered the original intervention,^32^ covering an introduction to CB principles, self-efficacy and managing groups, plus practise in CB techniques of formulation (linking thoughts, feelings and behaviours) and Socratic questioning (as opposed to didactic information giving).^25^ ^26^ RAFT sessions will be discussed and practised in groups, with input from patient partners, and observation and feedback from trainers. To complete their training, tutor pairs must deliver a practise course to patients not in the RCT, observed by a trainer, with feedback and debriefing after each session.

*Clinical supervision*: The tutors are experienced rheumatology clinicians, requiring only minimal clinical supervision/support in using CB approaches. Tutors will select one session in alternate courses for observation by the psychologist or OT with feedback and debriefing. Telephone support will be available (psychologist).

*Fidelity to RAFT (quality assurance)*: Fidelity will be monitored in one session of every course in each centre. An independent clinical psychologist will randomly select a session, and using a template, record use of CB approaches, adherence to session plans, use of RAFT materials and any unhelpful delivery styles (eg, didactic teaching). If fidelity is weak, clinical supervision is given for the next session, followed by a further independent observation.

### Usual care

The arthritis fatigue self-management booklet in common use^46^ was developed from this group's original RCT.^32^ It contains information on all the RAFT topics, a pull-out sample activity/rest chart to complete, and suggests that patients ask their rheumatology team for support with goal setting if necessary. This 32-page Arthritis Research UK free booklet is routinely provided by UK Rheumatology Units and discussed by nurses.

Usual care will be delivered to all patients at the baseline visit after consent and assessment, but prior to randomisation. The research nurse will spend 5–6 min discussing the booklet, using a standardised discussion guide, including that the booklet suggests patients might wish to request support from the rheumatology team. To minimise the risk of contamination between arms, the local nurse specialist managing such requests will try to not book a control patient in to see a clinician who is also an intervention tutor. This may be unavoidable in small teams, therefore, tutors will record any control patient appointments for fatigue support, for use in the analysis. Clinical care will continue as usual for both groups throughout; any medication changes will be recorded for potential subset analysis.

### Outcome assessments

Outcomes will be measured at Weeks 0, 6, 26, 52, 78 and 104 using measures validated in RA (table 2). The primary outcome is fatigue impact at 26 weeks, when patients should have become skilled at self-management. The 6-week assessment (posted 2 days after session 6) will capture the intense support of the weekly sessions, while weeks 52, 78 and 104 assessments will capture long-term outcome with skills either embedded or forgotten. For exploratory analysis of the week 14 consolidation session, fatigue data alone will be collected at weeks 10 and 18.

**Table 2:** Outcome measurement

Outcome	Scale (weeks 0, 6, 26, 52, 78, 104)
*Clinical status and quality of life*	
Fatigue impact*	Bristol RA Fatigue NRS Impact (BRAF-NRS)^40^ ^47^
Fatigue severity*	Bristol RA Fatigue NRS Severity (BRAF-NRS)^40^ ^47^
Fatigue coping*	Bristol RA Fatigue NRS Coping (BRAF-NRS)^40^ ^47^
Fatigue overall and subdimensions*	Bristol RA Fatigue Multi-Dimensional Questionnaire (BRAF-MDQ)^40^ ^47^
Mood	Hospital Anxiety and Depression scale^48^
Pain	Visual analogue scale
Quality of life	EQ-5D-5L;^49^ Global question from Arthritis Impact Measurement Scale^50^
Sleep quality	Single question from Pittsburgh Sleep Quality Index^51^
Leisure activities	Discretionary activity subscale of Valued Life Activities scale^52^
Social engagement	Increased seeking of social support (unvalidated question, weeks 52, 104)
Disability	Modified Health Assessment Questionnaire^54^
Disease activity DAS28^55^†	Protein, Patient global opinion (VAS)Painful joints and swollen joints (clinician assessed); C-Reactive
Disease activity PDAS2^56^	Patient Self-report Disease Activity Score (PDAS2)^56^
*Cost effectiveness*	
Utility scores	EQ-5D-5L;^49^ Work Productivity and Activity Impairment scale^57^
Costs—staff (tutors, trainers)	Time logs, travel forms, materials used (for training, practice and intervention courses); group sizes
Costs—NHS primary care	Patient questionnaires for medications, visits (excluding monthly blood monitoring for RA drugs)
Costs—NHS secondary care	In/outpatient episodes for rheumatology and orthopaedics, hospital transport (all via hospital computer)
Costs – patients	Patient questionnaires for travel related to healthcare, plus RAFT and sick leave where appropriate
*Processes for behaviour change*	
Helplessness, Self-efficacy	Arthritis Helplessness Index^58^ and RA Self-Efficacy scale^59^
*Acceptability and feasibility*	
RAFT and usual care (booklet)	Satisfaction (unvalidated question) Week 26 only
RAFT	Recommending the course to others (unvalidated question) Week 26 only
Feasibility of NHS delivery	Monitoring of course scheduling and delivery; tutor experiences via qualitative evaluation after cohort 4

*Also measured weeks 10 and 18, 4 weeks either side of consolidation session 7.

†Variables combined using algorithm to form DAS28^55^ measured at weeks 0 and 26 only as necessitates hospital visit.

RAFT, Reducing Arthritis Fatigue by clinical Teams.

Fatigue impact (primary outcome) and fatigue experiences will be measured using the trio of Bristol RA Fatigue NRS (impact, severity, coping) and multidimensional questionnaire (BRAF-NRS, BRAF-MDQ).^40^ ^47^ Other clinical, mood and quality of life variables, plus cost-effectiveness, process, acceptability and feasibility will be evaluated as described in table 2.^48–59^ The qualitative evaluation of the tutor experiences, will be by focus group and/or one-to-one interviews with tutors after their final RAFT course.^60^ ^61^ These will be conducted by a researcher not involved in training, to explore their experiences of RAFT training and delivery, including barriers and facilitators.

### Analysis plan

Quantitative analysis will be performed using Stata, blind to allocated arm, and will follow the Statistical and Health Economics Plan approved by the independent Trial Steering and Data Management and Ethics Committees (TSC, DMEC). To be defined as having received RAFT, a patient in the intervention arm must have attended at least session 1. Data entry checks against 100% of questionnaires will be made by two researchers together.

*Clinical status analysis*: The primary intention-to-treat analysis will involve between-arm comparisons for the primary outcome fatigue impact (BRAF-NRS Impact) at 26 weeks, adjusted for baseline values. Analyses of covariance (ANCOVA) will use multivariable linear regression models and standardised effect sizes calculated (adjusted mean difference divided by pooled baseline SD), with >0.5 considered a clinically meaningful effect. Sensitivity analyses will be conducted by (1) additional adjustment for any variables displaying imbalance at baseline, (2) fitting multilevel mixed effects models to investigate any clustering effect from delivery in groups and centres, and (3) multiple imputation techniques to investigate the impact of missing data, based on 20 imputed data sets, with baseline fatigue severity, impact, pain and disease activity added to the imputation model as variables predictive of missingness.^62^ Secondary outcomes will be analysed in the same way, including analysis of the four BRAF-MDQ fatigue subscales, and preliminary multivariable analysis of different attendance rates/patterns.

Further analyses using repeated measures mixed effects ANCOVA models will examine the effect of interventions over time by including up to four follow-up scores (26, 52, 78, 104 weeks) for the primary outcome, adjusting for baseline scores. Convergence/divergence between trial arms over time will be investigated by including appropriate interaction terms in the model. Clustering effects will be investigated by including CB group and centre IDs as additional levels. We will examine (and if necessary, adjust for) possible differences between arms for RA medication changes. Where numbers allow, we will explore coefficients of predictors of outcome.

*Cost effectiveness analysis*: Actual expenses of training and delivery will be recorded. Unit costs for NHS staff time for training and intervention delivery will be based on national estimates and costs of medications; community, primary and secondary care during follow-up will be based on national tariffs,^63–65^ supplemented by micro-costing or local estimates. Productivity costs due to RA and fatigue will be estimated based on average weekly earnings stratified by age.^66^ Resource use will be combined with unit costs to estimate the incremental cost or savings of the group CB programme over the 2-year period. The primary analysis will be from the societal perspective, including productivity costs. Secondary analyses will restrict the perspective to NHS and personal social services costs.

EQ-5D-5L utility scores will be used to estimate Quality Adjusted Life Years (QALYs), adjusting for any baseline imbalances.^67^ Missing data on costs or QALYs will be multiply imputed. Costs and outcomes during the second year will be discounted in line with NICE guidance.^68^ Cost and QALY data will be combined to calculate an incremental cost-effectiveness ratio (ICER) and net monetary benefit (INMB) statistic,^69^ which will indicate whether the group CB programme is cost-effective compared with NICE thresholds of £20 000–£30 000 per QALY gained. Uncertainty in the point estimate of cost per QALY will be quantified by using bootstrapping methods to calculate CIs around the ICER and INMB. The probability that the group CB programme is cost-effective will be depicted using a cost-effectiveness acceptability curve, and one-way sensitivity analyses used to judge potential impact of other sources of uncertainty.

*Analysis of tutor experiences*: An inductive thematic approach will identify themes grounded in the participants’ data.^70^ After reading and re-reading the anonymised transcripts, significant statements will be extracted, coded, explored for links, built into overarching themes, and exemplified by participants’ quotations. A second qualitative researcher will independently analyse a subset of the transcripts and themes compared and agreed. The focus group and the interview findings will be integrated to provide a comprehensive account of tutors’ experiences.

## Ethics and dissemination

*Ethics*: Patients will receive a detailed information sheet, and research nurses obtaining written consent will be trained in Good Clinical Practice. Patients may decline to participate, or withdraw at any time without affecting their clinical care, which will continue as normal during the trial. On the basis of the original RCT, no serious adverse events related to the intervention are anticipated, but any events will be reported in accordance with the sponsoring Trust's policy.

*Research governance*: The trial is sponsored by University Hospitals Bristol NHS Foundation Trust (ie, covered by NHS indemnity), managed by the RAFT central study team and supported by a Trial Management Group, TSC and DMEC. Data will be collected and retained in accordance with the Data Protection Act 1998 and anonymised during data entry. Study documents (paper and electronic) will be retained in a secure location for 5 years after trial completion.

*Patient and Public Involvement*: Two patient research partners (Robinson, Rooke), participants in the original RCT, are coapplicants and members of the Trial Management Group. They contributed to proposal development, particularly in elucidating the appropriate outcomes to assess questionnaire packages, information sheets and recruitment practicalities. They will help deliver tutor training, support interpretation of findings, and advise on implementation.

*Dissemination and implementation*: Dissemination to the clinical and academic community will be by academic papers, conference presentations and submission to NICE guidelines; to participants through a RAFT newsletter, and to the wider RA population through the National RA Society website and posters in the participating centres. The trainers’ experiences and supervision observations, and the qualitative evaluation with the tutors, will inform RAFT manual and training refinement, and practical methods of widespread implementation of RAFT across rheumatology (eg, training DVDs). RAFT training and materials will be subject to copyright and an appropriate royalty-free licensing regimen to maintain integrity.

## Discussion

Self-management of LTCs is a key government target, and while CBT interventions for fatigue are successful, they cannot be implemented in routine clinical care due to a shortage of clinical psychologists or CB therapists in usual clinical teams. However, in complex LTCs, nurses and occupational therapists have an understanding of the interactions between patients’ multiple and fluctuating symptoms, disease activity, and lifestyle. Clinical teams are thus well placed to deliver such an intervention, if they can be provided with skills in basic CB approaches and group management, and the materials needed to deliver the course. Many good interventions fail to translate from promising RCT data to clinical implementation because of practicalities. This current study's approach of skilling-up clinical teams has implications for facilitating interventions that can be delivered widely across the NHS in a cost-effective manner, and the principle could be applied to other symptoms in many LTCs. This trial is in progress and recruiting to time (June 2015, 22/28 planned CB courses randomised).
